# Bridging reward and resilience: the endocannabinoid system as a unifying mechanism in exercise-induced protection against major depressive disorder

**DOI:** 10.3389/fpsyt.2026.1766980

**Published:** 2026-03-09

**Authors:** Guanmin Zhang, Qiuju Hu, Haiyang Zou

**Affiliations:** 1 Department of Physical Education, Gangneung-Wonju National University, Gangneung, Republic of Korea; 2 Department of Physical Education, Henan Technical Institute, Zhengzhou, Henan, China

**Keywords:** ECS, major depressive disorder, motion, neuroplasticity, non-pharmacological interventions, reward system, stress resilience

## Abstract

Major depressive disorder (MDD) refers to a complex mental disorder defined by hindered reward system and hindered stress resilience. The limitations of traditional monoamine antidepressants have prompted the academic community to study new pathological processes and intervention strategies. Major depressive disorder arises from a complex interplay of psychological, social, and biological factors. Among the latter, dysfunction of the endocannabinoid system (ECS) has emerged as a critical pathological mechanism contributing to the core symptoms. This review demonstrates the key idea that exercise as a powerful non-pharmacological intervention can increase stress resilience and exert antidepressant effects by positively activating the ECS. Exercise, especially moderate intensity aerobic exercise, can significantly increase the levels of major endogenous cannabinoids AEA and 2-AG, and exert effects at multiple levels by activating CB1 receptors: at the acute level, it can immediately promote mood, generate analgesic effects and improve the termination of the stress response; At the long-term level, it can drive synaptic plasticity, facilitate hippocampal neurogenesis, and regulate neuroimmunity, thereby obtaining lasting structural improvement of emotional and stress neural circuits. These processes work together to reshape the brain’s reward function and establish internal resilience against stress. In comparison to drug therapy, ECS-regulated exercise interventions have the unique benefits of high safety, systemic advantages, and endogenous reward reinforcement. Thus, individualized exercise therapy for ECS represents a promising mechanism-induced non-pharmacological intervention approach offering a new aspect and perspective for the prevention and rehabilitation of depression.

## 1 Introduction

Major depressive disorder (MDD) is a general mental disorder with a high disability rate, and its common pathological features comprise persistent low mood, loss of interest, anhedonia, and cognitive impairment, which places a heavy burden on patients and society (1). The research and treatment approaches of its pathophysiological mechanism primarily center on the dysregulation of monoamine neurotransmitters, such as serotonin, norepinephrine and dopamine (2). However, a considerable number of patients have poor response, delayed onset, or difficulty tolerating the side effects of monoamine antidepressants, prompting us to investigate the more complex neurobiological underlying depression (3). However, contemporary understanding frames MDD not as a simple neurotransmitter deficiency, but as a heterogeneous disorder stemming from a complex bio-psycho-social interplay, where genetic vulnerability, chronic stress, adverse life events, and maladaptive cognitive patterns converge to disrupt the homeostasis of multiple neural systems (4, 5).

Among these disrupted systems, the endocannabinoid system (ECS), as an evolutionarily highly conserved lipid signaling system, has gained prominence for its integral role in regulating emotional, stress, and reward processing (6). The ECS functions not only as a reward system implicated in pleasure generation but also as a central regulator maintaining the dynamic balance of neural circuits. Its dysfunction directly leads to an imbalance within the emotion, reward, and stress-coping networks, hence forming the pathological basis of depression (7). A large body of preclinical and clinical evidence reveals that a depressive state is strongly linked to a state of endocannabinoid deficiency, manifested by decreased levels of major endocannabinoids (such as AEA and 2-AG) in the brain and decreased signaling function of cannabinoid receptors (8). Conversely, A well function ECS is considered a key molecular cornerstone of stress resilience, which refers to an individual’s ability to maintain mental health in adversity (9).

In this context, exercise has been extensively indicated to have antidepressant and emotional resilience effects. It is interesting that these advantageous effects of exercise are tightly linked to its positively activation of the ECS (10). Research has shown that exercise, especially aerobic exercise, can significantly increase the levels of endogenous cannabinoids in the circulatory system and central nervous system (CNS), which offers a direct molecular mechanism to clarify post-exercise pleasure and mood enhancement (11). More significantly, exercise-driven ECS activation can not only bring immediate emotional regulation, but also obtain long-term improvement of brain structure and function by promoting neuroplasticity and regulating neuroimmunity, thereby fundamentally increasing the resilience of individuals to cope with stress (12).

However, there is a conceptual divide in the mechanistic explanation of the benefits of exercise: its acute rewarding effects (such as ‘runner’s high’) are primarily associated with the mesolimbic dopamine system (13) (14), while its long-term antidepressant and resilience-enhancing effects rely more on mechanisms such as brain-derived neurotrophic factor (BDNF)-mediated neuroplasticity, hippocampal neurogenesis (15), hypothalamic-pituitary-adrenal (HPA) axis normalization (16, 17), and anti-inflammatory effects (18, 19). This raises a key question: is there an intrinsic connection between the immediate rewards generated by exercise and its long-lasting therapeutic benefits? This review proposes a core argument: the endocannabinoid system (ECS) serves as a crucial molecular bridge connecting these two temporal effects (20). We hypothesize that exercise, as a potent physiological stimulus, simultaneously activates two complementary pathways by upregulating AEA and 2-AG levels: (1) Exercise-induced ECS activation enhances dopamine signaling by acting on CB1 receptors in reward-related brain regions such as the ventral tegmental area (VTA) and nucleus accumbens (NAc) (13, 21). This directly induces pleasure (11) and positively reinforces exercise motivation, providing the necessary behavioral foundation for maintaining long-term exercise intervention. (2) Similarly, exercise-driven ECS activation promotes BDNF release, synaptic plasticity (22), neurogenesis, and effectively inhibits HPA axis overactivation (17) and neuroinflammation (18, 23) by acting on CB1/CB2 receptors in brain regions such as the hippocampus, prefrontal cortex, and amygdala. These structural adaptations serve as the neurobiological basis for enhanced stress resilience and sustained relief of depressive symptoms. Therefore, the ECS does not function solely as a ‘reward system’ or ‘antidepressant system’, but rather as a multifunctional homeostatic regulatory center (7), coupling the immediate emotional enhancement of exercise with its long-term neurorestorative and resilience-building functions (24).

Accordingly, this review aims to explore this integrative hypothesis. We first examine the dual role of the ECS in depression pathophysiology and stress resilience formation. We then detail the effects of exercise on various ECS components and its subsequent impact on the brain’s reward system and neuroplasticity. Based on this, we will delve into the acute rewarding effects and long-term neural adaptive effects mediated by ECS, respectively, and elucidate how they synergize. Finally, we evaluate the potential, advantages, and prospects for individualized application of ECS-based exercise interventions in clinical depression management, along with future research directions. Through this synthesis, we aim to provide a robust theoretical framework that reconciles the acute rewarding and long-term therapeutic effects of exercise, offering a novel and cohesive perspective on exercise as a mechanism-informed intervention in mental health.

## 2 ECS and depression: from homeostasis regulators to pathological hubs

**Figure 1:**
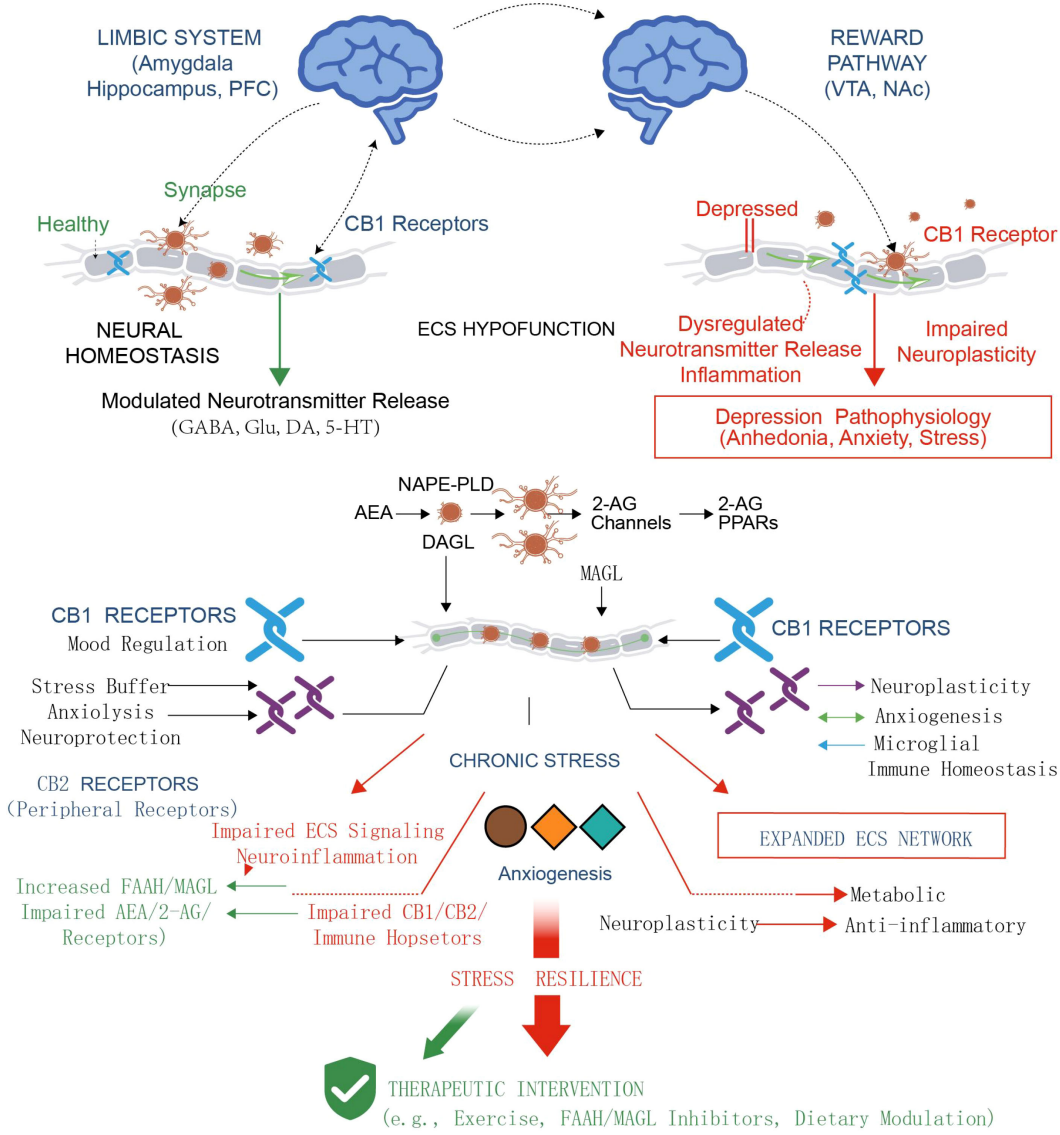
The transition of ECS from a homeostatic regulator to a pathological hub in depression and the restorative role of therapeutic interventions. This figure summarizes the multi-level impact of ECS dysfunction in depression and the reversal mechanism by therapeutic interventions. Upper Panel (Neural Circuit Level): Contrasts Neural Homeostasis (Left) with ECS Hypofunction (Right). In the healthy state, functional CB1 receptors precisely modulate synaptic neurotransmitter release (GABA, Glu, DA, 5-HT) within the Limbic System and Reward Pathway. Conversely, ECS hypofunction in the depressed state leads to dysregulated neurotransmission, inflammation, and impaired neuroplasticity, resulting in pathophysiology such as anhedonia, anxiety, and stress. Lower Panel (Molecular and Systemic Level): Illustrates the divergence under Chronic Stress. The pathological pathway (Left side) is characterized by impaired ECS signaling (e.g., increased FAAH/MAGL activity, impaired AEA/2-AG levels) and dysfunction of CB1/CB2 receptors, driving neuroinflammation and anxiogenesis. The intervention pathway (Right side and Bottom) demonstrates how Therapeutic Interventions (e.g., Exercise, Dietary Modulation, FAAH/MAGL Inhibitors) promote Stress Resilience. These interventions activate the “Expanded ECS Network,” restoring immune homeostasis and enhancing neuroplasticity and metabolic function, ultimately reversing the stress-induced deficits. ECS, Endocannabinoid System; AEA, Arachidonic acid ethanolamine; 2-AG, 2-arachidonic glycerol; CB1, Cannabinoid receptor type 1; GABA, Gamma-aminobutyric acid; Glu, Glutamate; DA, Dopamine; 5-HT, Serotonin; FAAH, Fatty acid amide hydrolase; MAGL, Monoacylglycerol lipase.

For a long time, research on the pathophysiological processes of MDD has primarily centered on the dysregulation of monoamine neurotransmitters (2). Nevertheless, there is increasing evidence that the ECS is a neuromodulatory system that is vital in depression, but has long been underestimated, ECS is not just a pleasure-producing reward system, It is also a key regulator that sustains the dynamic balance of neural circuits (7, 25). Its dysfunction directly causes imbalances in the emotion, reward, and stress coping networks, hence forming the pathological basis of depression (7). **Figure 1** shows The role of ECS as a steady-state regulatory hub in the pathology of depression and exercise induced stress resilience.

### 2.1 The biological basis of the ECS: a complex signaling network that transcends a single receptor

The ECS is an evolutionarily highly conserved lipid signaling system with key components, such as endocannabinoid ligands, their synthesis and degrading enzymes, and cannabinoid receptors (26).

#### 2.1.1 Core endocannabinoid ligands: AEA and 2-AG

Its main ligands are arachidonic acid ethanolamine (AEA) and 2-arachidonoylglycerol (2-AG) (27). Unlike classical neurotransmitters, AEA and 2-AG are lipid mediators that are synthesized and released on demand and act retroactively on cannabinoid receptors at the presynaptic terminal after postsynaptic neuronal generation, thereby precisely modulating neurotransmitter release (28). This transient retrograde signaling is a key mechanism by which the ECS sustains the stability of the neuronal microenvironment.

Although both AEA and 2-AG activate CB1 and CB2 receptors, they have profound functional differences that constitute a functional division within the ECS. AEA has a higher affinity for CB1 receptors and plays a transient tuning role primarily in mood, stress, and fear subsidence. Rezende pointed out that both AEA and 2-AG are synthesized on demand and have short half-lives (28). Inhibition of its degrading enzyme FAAH has been reported to increase fear subsidence and decrease the adverse effects of chronic stress, stressing the crucial position of AEA in adaptive emotion modulation (29). Conversely, 2-AG is the most abundant endocannabinoid in the brain, and as a complete agonist of CB1 receptors, it plays a wider role in suppressing neurotransmitter release (30). Current literature has suggested that 2-AG is vital in neuroinflammation and neuroplasticity via the modulation of its degrading enzyme MAGL. Inhibition of MAGL can significantly enhance 2-AG levels, which in turn drives the expression reprogramming of immune and inflammation-associated genes in microglia and astrocytes, generating strong anti-inflammatory and neuroprotective effects (18). This indicates that 2-AG is an important bridge connecting neural activity and immune response.

#### 2.1.2 Classical cannabinoid receptors: CB1 and CB2

##### 2.1.2.1 CB1 receptors: ubiquitous neuromodulators

The function of the ECS is spatially notable via its receptors. CB1 receptor is one of the most abundant G-protein-coupled receptors in the CNS, and its expression is highly enriched in brain regions that are tightly linked to mood, cognition, and reward, such as the neocortex, hippocampus, amygdala, and basal ganglia (31). Mackie elaborates further, indicating that this G-protein-coupled receptor has a characteristic distribution in the nervous system: it is notably abundant in the cortex, hippocampus, amygdala, basal ganglia outflow pathways and cerebellum, primarily on neurons, stressing the significant role of this receptor in modulating specific synaptic neurotransmission (32). Traditionally, CB1 receptors are primarily expressed on GABAergic interneurons and modulate neural circuits via inhibitory input (33). Nevertheless, a breakthrough study pointed out that CB1 receptors are also expressed in a subset of neurons that co-release dopamine and glutamate in the ventral tegmental region of the midbrain (21). This discovery fundamentally alters our understanding of the effects of cannabinoids: they not only indirectly modulate, but also directly modulate the excitability and reward signal output of dopaminergic neurons.

##### 2.1.2.2 CB2 receptors: neuroimmune interface

Meanwhile, CB2 receptors, which were previously thought to be predominantly present in peripheral immune cells, have been determined to exist in microglia and specific neuronal subpopulations of the CNS (34). Its role in the nervous system primarily impacts mood and behavior by modulating neuroimmunity. CB2 receptor activation exerts strong anti-inflammatory effects and is considered a key player in modulating microglial activity, positioning it at the intersection of stress, neuroinflammation, and depression (35).

#### 2.1.3 Expanded ECS receptor network: GPR55, TRPV1, and PPARs

##### 2.1.3.1 GPR55: an atypical cannabinoid receptor

Recent evidence underscores the important roles of other ECS-associated receptors beyond CB1 and CB2. GPR55 is recognized as an “atypical” cannabinoid receptor. Its activation can enhance neurotransmitter release at central synapses via distinct Ca^2+^ signaling pathways, implicating it in the regulation of neural excitability and synaptic plasticity (36). Furthermore, GPR55 activation exhibits neuroprotective effects, including promoting hippocampal neurogenesis and suppressing neuroinflammation, suggesting its potential role in mood regulation (37).

##### 2.1.3.2 TRPV1: the ionotropic target and bidirectional modulator

The transient receptor potential vanilloid 1 (TRPV1) channel serves as an “ionotropic” target for AEA, playing a well-established role in nociception and thermosensation (38). Critically, in emotional regulation, AEA exhibits a bidirectional effect: at low concentrations, it primarily activates CB1 receptors to produce anxiolytic effects, while at higher concentrations or under specific conditions, it can activate TRPV1 to promote anxiogenic responses (39). This balance between CB1 and TRPV1 signaling is crucial for understanding ECS-mediated emotional homeostasis.

##### 2.1.3.3 PPARs: nuclear receptors for metabolic and inflammatory regulation

Furthermore, endocannabinoids like AEA and 2-AG can also act as ligands for peroxisome proliferator-activated receptors (PPARs), particularly PPARγ. PPARγ is a nuclear receptor involved in regulating energy metabolism and exerts potent anti-inflammatory and neuroprotective effects, providing another significant pathway through which the ECS influences systemic and cerebral energy homeostasis (40).

#### 2.1.4 Metabolic enzymes: biosynthesis and Degradation

The spatiotemporal precision of ECS signaling is tightly governed by key metabolic enzymes. Biosynthesis of AEA and 2-AG is primarily catalyzed by N-acyl-phosphatidylethanolamine-specific phospholipase D (NAPE-PLD) and diacylglycerol lipase (DAGL), respectively. Their degradation is predominantly mediated by fatty acid amide hydrolase (FAAH) for AEA and monoacylglycerol lipase (MAGL) for 2-AG (41). The activity of these enzymes directly dictates the local concentration and signaling duration of endocannabinoids, making them critical targets for pharmacological intervention and functional regulation of the ECS (42).

#### 2.1.5 Integrated network function

Thus, the ECS constitutes a complex signaling network via the spatiotemporal-specific effects of its two primary ligands, the broad distribution of multiple receptors (CB1, CB2, GPR55, TRPV1, PPARs) on central neurons, glia, and immune cells, and the fine-tuned control by metabolic enzymes (43, 44). Adding an extra layer of complexity to these interactions, the ECS uniquely impacts the neurotransmission of dopamine and serotonin (13). It finely modulates mood, motivation, and reward by modulating the release of monoamine transmitters, such as dopamine, norepinephrine, and serotonin (45). More significantly, in the stress response, the ECS is a key regulator of neuroplasticity, and its signal modifications directly cause modifications in synaptic function, aiding the brain adapt to environmental challenges and sustain homeostasis (46, 47).

### 2.2 ECS dysfunction: homeostasis failure in depression

Given the central role of the ECS in sustaining neural homeostasis, its dysfunction is naturally tightly linked to the pathophysiology of depression. Substantial evidence from both preclinical and clinical studies has established that a deficit in endocannabinoid signaling represents a key etiological factor in depression, positioning the pharmacological augmentation of this system as a novel therapeutic target (48). A large amount of preclinical and clinical evidence suggests that a depressive state is associated with a broader endocannabinoid deficiency, encompassing not only the classic ligands and CB1 receptors but also dysregulation within the expanded ECS network.

#### 2.2.1 Classic ECS deficiency: ligand and CB1 receptor hypofunction

Patients with depression or animal models of chronic stress commonly exhibit decreased levels of AEA and/or 2-AG in the brain, as well as decreased signaling function of CB1 receptors (46). This core deficiency can trigger a multifaceted chain reaction disrupting key neural circuits. In reward centers, such as the nucleus accumbens, the weakening of AEA signaling decreases its capacity to inhibit GABAergic interneurons via CB1 receptors, leading to reduced dopamine release. This is directly manifested as anhedonia—a core symptom of depression (49). Furthermore, the hindered function of CB1 receptors located directly on dopaminergic neurons in the ventral tegmental area may directly blunt reward signal output and motivation (21). The ECS is a critical stress buffer. In the amygdala, basal AEA levels tonically inhibit excitatory glutamatergic transmission via CB1 receptors. Chronic stress induces a persistent reduction of AEA in this region, diminishing this inhibitory brake. This leads to amygdala hyperexcitability and uncontrolled activation of the hypothalamic–pituitary–adrenal (HPA) axis, resulting in persistent anxiety-like behavior and maladaptive fear memories (29). Attenuation of 2-AG signaling compromises its potent anti-inflammatory and neuroprotective effects, which are mediated through CB1 and CB2 receptors on microglia and astrocytes (18). This impaired ECS tone makes the brain more susceptible to neuroinflammation, a significant pathogenic driver of depression (19).

#### 2.2.2 Dysregulation of the expanded ECS network

Emerging evidence indicates that the pathophysiology of depression involves more than just the classic AEA/2-AG-CB1 axis; it extends to other ECS-associated receptors and enzymes. The role of CB2 receptors in neuroinflammation positions them centrally in depression pathology (35, 50). While CB2 expression in the brain is generally low under homeostatic conditions, its regulation is highly dynamic and context-dependent (51). In depression, often characterized by a state of chronic low-grade neuroinflammation, the expected upregulation of CB2 as an adaptive, anti-inflammatory response may be impaired or dysregulated (52, 53). This failure to adequately engage the CB2-mediated neuroimmune regulatory pathway likely exacerbates inflammatory processes and contributes to disease progression (35).

The delicate balance between CB1-mediated anxiolysis and TRPV1-mediated potential anxiogenesis is crucial for emotional homeostasis. In depression, a deficiency in AEA signaling may disproportionately favor TRPV1-mediated pathways or alter the activation threshold of TRPV1, disrupting this balance. This shift could contribute to the heightened anxiety and negative affective states commonly observed in MDD (7, 54). The observed deficiencies in AEA and 2-AG levels are likely driven by alterations in their metabolic enzymes. Increased activity of degradative enzymes like fatty acid amide hydrolase (FAAH) and monoacylglycerol lipase (MAGL), or decreased activity of synthetic enzymes (e.g., NAPE-PLD, DAGL), can lead to a rapid clearance and reduced bioavailability of endocannabinoids (55). This enzymatic dysregulation represents a key upstream pathological mechanism leading to endocannabinoid tone deficiency.

### 2.3 ECS: The intrinsic molecular cornerstone of stress resilience

Conversely to the pathological model described above, a well-functioning and adaptive ECS is the molecular cornerstone of stress resilience—an individual’s ability to sustain mental health in the face of adversity. Resilience is not just the absence of pathological changes, but an active adaptation process (9, 56). A responsive ECS system capable of rapidly mobilizing AEAs and 2-AGs when stress strikes, performing his, neural tuning, functioning: suppressing excessive excitotoxicity, increasing protective inhibition, and regulating inflammatory responses (23, 57).

For instance, in acute stress, a momentary increase in AEA may aid limit over reactivity in the HPA axis and improve swift emotional stabilization (46, 58). Long-term adaptive training may increase the tension of the entire ECS (such as receptor expression, signal efficiency), so that the brain has a stronger buffering ability in the face of future challenges (29), It is worth noting that increasing ECS function, such as enhancing AEA levels via drug inhibition of FAAH, has been reported to improve the fading of fear memories, a significant indicator of resilience behavior (29). Likely, evidence has reported that activating CB1 receptors can avoid acute stress-driven anxiety-like behavior, oxidative stress and GABA reduction (59).

### 2.4 ECS and depression

Depression is a complex psychiatric disorder linked to emotional, cognitive, and physiological dysfunction, defined by persistent low mood, loss of interest, feelings of exhaustion, and social isolation (1). Dysfunction of the ECS is tightly linked to the occurrence and progression of depression, and the ECS may is vital in the pathological process of depression by modulating key neurobiological mechanisms, such as neurotransmitter balance, neuroplasticity, and immune homeostasis (50).

People with depression commonly display a phenomenon of ECS hypofunction, a condition known as endocannabinoid deficiency. This is manifested by a decrease in endocannabinoid levels, notably in the concentration of AEA and 2-AG (8). This weakening of endocannabinoid signaling can cause hindered neuroplasticity and mood regulation, which can boost symptoms of depression. Research has shown that the ECS (ECS) has a strong impact on nerve conduction, leading to changes in the concentration of endocannabinoids and their discovery in the anterior lobe fibers of patients with depression (60). Furthermore, the 2-AG signaling pathway is linked to depression susceptibility, which indirectly encourages the hypothesis that a reduction in 2AG concentration may cause neuroplasticity and emotion modulation disorders, thereby boosting the assertion of depressive symptoms (61).

The functional abnormalities of the ECS are not limited to the ligand level, but also the expression and function of its receptors have also changed significantly. Besides modifications in endocannabinoid concentrations, In the anterior lobe membrane of depressed patients, differences in endocannabinoid concentrations, binding affinity of CB1 receptors, and their density have been found (60). Meanwhile, the expression of CB2 receptors in immune cells is also linked to the pathophysiology of depression (35, 62). Among them, the decrease in the proportion of FoxP3 ^+^ CB1 ^+^ cells and CD3 ^+^/CD4 ^+^ CB2 ^+^ cells may lead to an imbalance in the immune homeostasis of patients with severe depression; and the increase in the proportion of CD20 ^+^ CB2 ^+^ cells may have an impact on the pathophysiological processes of severe depression (63). This reveals that the dysregulation of CB1 and CB2 receptors on specific lymphocyte subsets may be involved in the pathogenesis of depression by disturb immune homeostasis.

Given the tight association between ECS dysfunction and depression, modulating ECS activity via medication or behavioral intervention has become a promising therapeutic direction. Basic research confirms, endocannabinoid signaling dysfunction may cause depression-like behavior; Hence, enhancements in endocannabinoid signaling may represent a new treatment for depression control (60). One of the key processes of its therapeutic potential lie in the powerful regulatory ability of the ECS on neuroplasticity. The ECS impacts several functions of the brain, including neuroplasticity (24). Hence, increasing ECS function can not only promote symptoms, but also improve the repair and remodeling of damaged neural circuits from the root, offering a biological basis for the recovery of depression.

### 2.5 ECS-regulated stress resilience

Stress resilience is an individual’s ability to sustain dynamic adaptation and quickly return to homeostasis when facing psychological and physical stress, This ability is not simply to prevent stress responses, but to illustrate positively neurobiological adaptability in stressful challenges (64). Substantial evidence reveals that the ECS is a key molecular established block for comprising this stress resilience (46, 65). ECS offers an intrinsic buffer mechanism for key emotional and stress brain circuits by finely modulaying hypothalamic-pituitary-adrenal axis activity, modulating limbic system neurotransmitter release, and promoting neuroplasticity, thereby increasing the individual’s overall adaptability (17, 66).

#### 2.5.1 The key role of ECS in stress response

The ECS, notably signal transduction mediated by CB1 receptors, is crucial for initiating and terminating brain stress responses. Literature has confirmed that maintaining strong endogenous cannabinoid/CB1 receptor signaling is key to resisting stress-related pathological processes (67, 68). Therefore, substances that can effectively maintain this signaling pathway will help avoid or alleviate stress-related diseases. This view is strongly encouraged by cutting-edge research. A study suggested that high expression of CB1 receptors in astrocytes in the nucleus accumbens shell region, notably at the end foot site surrounding blood vessels, is linked to resilience in adult male mice under chronic social stress (69). Virus-induced astrocyte Cnr1 gene overexpression in this brain region not only generates baseline anxiolytic effects, but also dampen stress-driven anxiety and depression-like behaviors (69). Its mechanism is linked to promoting vascular-associated gene expression, decreasing the inflammatory response and morphological modifications in astrocytes after immune challenge. The study concludes, Astrocyte cannabinoid receptors1 improve resilience by suppressing stress-driven modifications in the blood-brain barrier (69). This reveals that the ECS, even CB1 receptors on non-neuronal cells, offer entirely new cellular and molecular processes for stress resilience by sustaining blood-brain barrier integrity and suppressing neuroinflammation.

#### 2.5.2 Neural circuit basis of stress resilience and emotion modulation

The ECS is able to precisely manage emotional and stress behavior outputs by modulating a wide scope of neural circuits in response to acute and chronic stress (46). Its bidirectional modification effect on the HPA axis is the key embodiment of its steady-state maintenance (16). Literature suggests that endogenous cannabinoid signaling is involved in activating and terminating the hypothalamic pituitary adrenal axis in response to acute and repetitive stress responses (16). This precise modulation of the neuroendocrine system, combined with the swift regulation of synaptic transmission by the ECS in brain regions, such as the amygdala, prefrontal cortex, and hippocampus, constitutes the neural basis of emotional stability (70, 71).

#### 2.5.3 Enhancing ECS function as a therapeutic approach to improve resilience

Given the key role of the ECS in stress response and emotion modulation, increasing ECS function is regarded as a possible approach to aid individuals better modulate their emotions and decrease symptoms of depression, thereby promoting their ability to cope with stress and resilience. Enhancements in endocannabinoid signaling may represent new treatments for depression control (60). Moreover, evidence suggests that dysfunction of endocannabinoid signaling may lead to depressive like behavior; Therefore, enhancing endogenous cannabinoid signaling may represent a new approach for the treatment of depression (72). This provides a theoretical basis for intervening in ECS through medication or behavioral means to improve stress resilience. Although there are currently two pathways for the development of cannabinoid drugs (plant extraction and single molecule synthesis), existing research still confirms the broad prospects of therapeutic development for ECS (73). Therefore, a well-functioning and adaptive ECS is the intrinsic biological basis of stress resilience, and intervention approaches for this system, notably non-pharmacological interventions, such as exercise, are expected to establish a more resilient brain state by strengthening its function.

## 3 Effects of exercise on the ECS

### 3.1 The regulatory effect of exercise on the ECS

**Figure 2:**
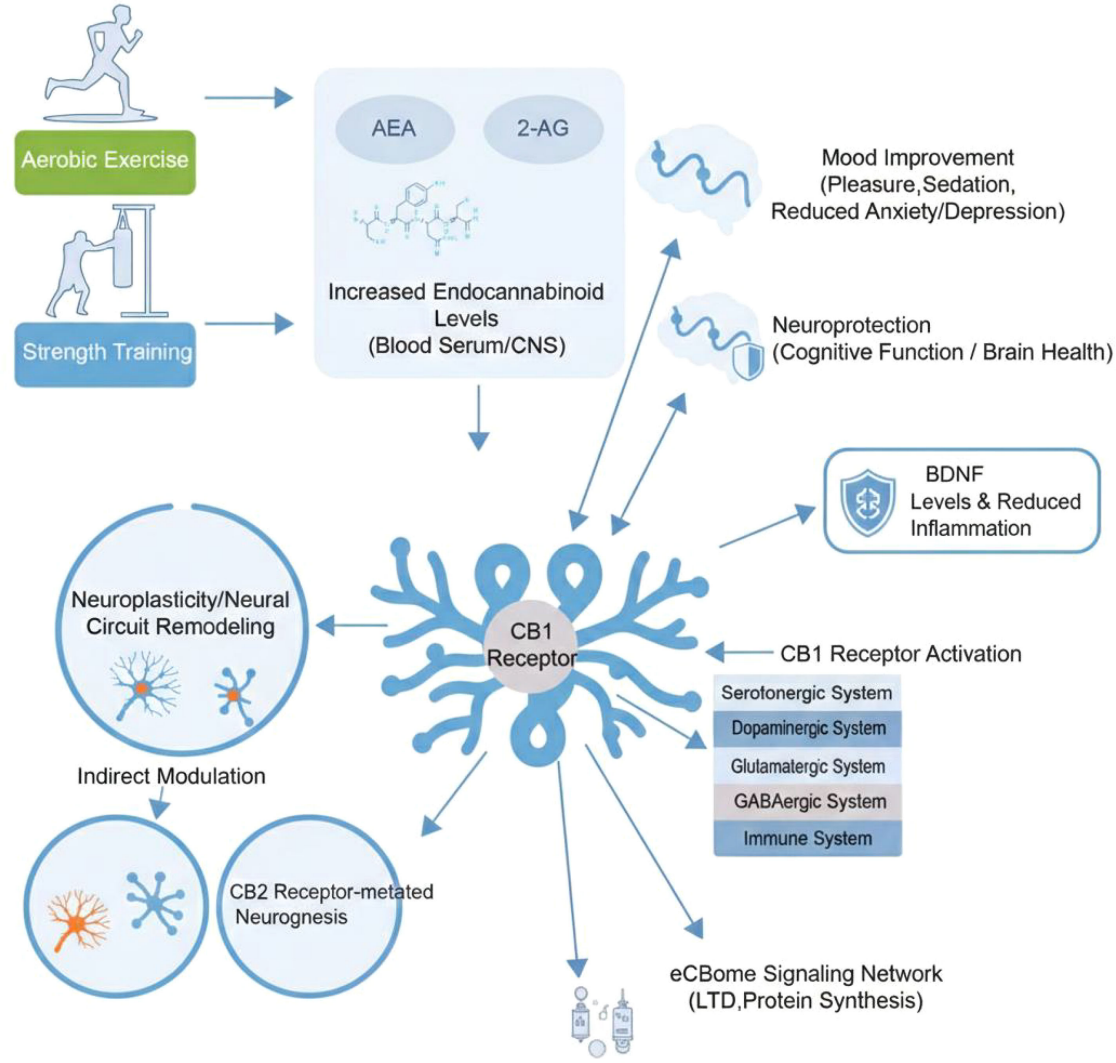
Multilevel mechanism of exercise regulating the endogenous cannabinoid system to produce antidepressant and neuroprotective effects. This figure illustrates how physical exercise (aerobic and strength training) modulates the ECS at multiple levels to promote mental health and neural resilience. Exercise triggers a significant elevation of endocannabinoids (AEA and 2-AG) in both the peripheral circulation and the central nervous system (CNS). These elevated ligands primarily activate CB1 receptors, which subsequently modulate key neurotransmitter systems, including the dopaminergic and serotonergic pathways, to enhance mood and motivation. Downstream signaling through CB1 receptors facilitates long-term synaptic depression (LTD) and protein synthesis, leading to neural circuit remodeling. Simultaneously, CB2 receptor-mediated pathways are essential for promoting hippocampal neurogenesis and increasing BDNF levels. Through the expanded eCBome signaling network (involving interaction with the immune system and reduced neuroinflammation), these combined processes result in sustained antidepressant effects, cognitive enhancement, and neuroprotection against stress-induced deficits. ECS (Endocannabinoid System), AEA, Anandamide; 2-AG, 2-Arachidonoylglycerol; CNS, Central Nervous System; CB1, Cannabinoid receptor type 1; CB2, Cannabinoid receptor type 2; LTD, Long-term depression; eCBome, Endocannabinoidome.

As a powerful non-pharmacological intervention, the effects of exercise on physical and mental health have been extensively indicated, and the neurobiological processes behind it are gradually being suggested (74). Among them, modulation of the ECS is regarded as a key link (74). A large number of evidence has reported that exercise can significantly alter the activity of the ECS, which is tightly linked to psychological experiences, such as pleasure, sedation and emotional improvement after exercise, offering a direct molecular explanation for the antidepressant effects of exercise and promoting stress resilience (10, 75). **Figure 2** demonstrates Multi level mechanism of exercise regulating the endogenous cannabinoid system to produce antidepressant and neuroprotective effects.

#### 3.1.1 Modifications in AEA and 2-AG levels and their biological significance

Exercise, especially aerobic exercise and endurance training, is a powerful physiological stimulus to enhance peripheral and central endogenous cannabinoid levels. This view is encouraged by cross-species studies. The latest research displays that exercise enhances the concentration of endocannabinoids in the blood serum, which offers a potential explanation for several of these changes (76). A thought-provoking comparative study further demonstrates that both humans and running dogs exhibit a significant increase in endogenous cannabinoid signaling in the circulatory system after high-intensity endurance running (77). This effect was not uncovered after low-intensity walking, and ferrets from non-runners cannot display a significant improvement in eCB signal after any intensity of exercise. The study linked exercise-driven eCB signaling to runners’ orgasm and habitual endurance exercise motivation, revealing that neurobiological rewards may clarify differences in habitual exercise activity and performance among mammals (77). Hence, exercise-driven enhancements in AEA and 2-AG levels are not only transient biochemical phenomena, but may also be the key biological basis for inducing positive emotions, relieving stress and anxiety, and supporting maintained exercise behavior. This exercise-driven enhancement of eCB signaling is closely correlated with improvements in subjective emotional states. Research has shown that the increase in AEA levels in the circulation after acute exercise is significantly associated with positive changes in emotional states, such as reduced anxiety and depression, and increased vitality (78).

#### 3.1.2 Correlation between CB1 receptor and neurotransmission

Exercise-triggering endocannabinoids exert their emotion-modulating and neuroprotective effects mainly by activating extensively distributed CB1 receptors (11, 79). CB1 receptors play a complex regulatory role in the CNS, and their signaling has been assessed in their regulatory role in states linked to mental illness (80). By activating CB1 receptors, enhanced eCB after exercise can significantly impact the monoamine neurotransmitter system, which is tightly linked to reward and mood (81). For instance, Adding an extra layer of complexity to these interactions, the ECS uniquely impacting the neurotransmitters of dopamine and serotonergic (20). This implies that exercise may indirectly improve the function of dopaminergic reward circuits and serotonergic emotion modulation systems via eCB signals, causing antidepressant and motivational effects.

#### 3.1.3 Motor, ECS and neural circuit remodeling

More significantly, exercise via CB1 receptor-induced effects expand beyond acute neurotransmitter modulation to the long-term neuroplasticity level (22, 82). Emerging research highlights a critical role for cannabinoid type 2 receptors (CB2Rs) in mediating the pro-neurogenic effects of exercise. Studies using *in vitro* models mimicking exercise conditions demonstrate that CB2Rs are required for the proliferation and early neuronal commitment of hippocampal neural progenitor cells promoted by exercise-associated factors (83). Furthermore, *in vivo* studies in chronically stressed animals reveal that modulating CB2R activity can shape the beneficial effects of physical exercise on adult hippocampal neurogenesis, inflammation, and BDNF levels, thereby enhancing stress resilience (84). At the synaptic level, activation of CB1 receptors can boost durable functional and structural modifications. A study of long-term depression (iLTD) targeting inhibitory synaptic transmission uncovered that, CB1-iLTD relies on protein synthesis and ubiquitination to drive structural modifications that decrease GABA release in the long term (85). This reveals that the exercise-driven eCB-CB1 signaling pathway can persistently promote interneuronal communication and reshape neural circuits by causing protein synthesis and ubiquitination-dependent presynaptic remodeling. This structural plasticity is fundamental for the brain to adapt to environmental challenges and repair functional deficits. Moreover, the benefits of exercise may be determined via a wider signaling network, the endocannabinoid group (eCBome), which appears to play a significant role in the correlation between exercise and neurological health, and may play a significant role in exercise-driven central and peripheral adaptive mechanisms, thus benefiting mental health (86).

#### 3.1.4 potential modulation of extended ECS network through exercise

Although the acute and long-term benefits of exercise are closely related to the regulation of classic endocannabinoid signals through CB1 and CB2 receptors, new evidence suggests that exercise may also interact with a broader and broader network of endothelial cells, including nonclassical cannabinoid related targets, such as GPR55, TRPV1 and PPAR, to promote emotional homeostasis and resilience in patients with depression.

Although direct evidence linking exercise to these targets is still limited in the context of depression, reasonable mechanisms can be proposed based on their known functions. For example, studies have shown that exercise can significantly improve the level of endogenous cannabinoid AEA in plasma, which is related to the sense of pleasure, anxiety relief and stress reconstruction after exercise (77), while AEA plays different biological roles mainly through CB1 and TRPV1 receptors in the low/high concentration range, suggesting that there is a key balance regulation mechanism between AEA CB1 and AEA TRPV1 signals (87); Excessive or long-term stress (aimed at counteracting exercise) may shift AEA to TRPV1 activation, leading to anxiety like state (88, 89). Therefore, exercise can help restore this balance, although this hypothesis needs to be directly tested.

Similarly, the metabolic and anti-inflammatory effects of exercise may be related to PPAR γ, a nuclear receptor activated by endogenous cannabinoids such as AEA and 2-AG. Regular physical activity can improve systemic metabolism and reduce low-grade inflammation, both of which are not regulated in depression (90, 91). PPAR γ activation has strong anti-inflammatory and neuroprotective effects (92). It is suggested that exercise may indirectly enhance the PPAR γ signal by increasing the tension of endogenous cannabinoid or through other exercise-induced mediators (such as adiponectin), thus helping to alleviate the neuroinflammation in patients with depression.

GPR55 is an atypical cannabinoid receptor, which is involved in neurogenesis and synaptic plasticity, and is another potential interface (36, 93). Exercise is a powerful inducer of hippocampal neurogenesis and BDNF release. Whereas GPR55 activation promotes neurogenic and anti-inflammatory effects (36, 93) it is conceivable that exercise may regulate GPR55 signal directly through ligand availability or indirectly through neurotrophic cross-talk to promote structural plasticity. However, there is a lack of clear research on the interaction between exercise and GPR55.

In a word, although the classic cb1/cb2 axis is still the most characteristic pathway, the ECS network expanded through GPR55, TRPV1 and PPARs provides additional and unexplored mechanisms through which exercise may play an antidepressant and restorative role. Future research should focus on the empirical verification of these interactions, especially in the preclinical model of depression, to fully clarify the comprehensive role of endothelial cells in exercise-induced neuroprotection.

### 3.2 Exercise type and ECS regulation

The regulation of the endocannabinoid system (ECS) by exercise is not a homogenous process. Different types of exercise, primarily divided into aerobic endurance exercise and strength training, may affect ECS activity and function through various pathways, thereby promoting mood improvement and neural health in multiple ways. Recognizing these differences is crucial for advancing precise exercise intervention (94). Aerobic exercise has been widely proven to be a potent stimulus that can directly elevate endogenous cannabinoid levels. Exercise modulation of the ECS is not a homogenized process, and various types of exercise – primarily divided into aerobic exercise and strength training – may impact ECS activity and function via various mechanisms, thereby promoting mood improvement and neurological health in a variety of ways (95). Recognizing these differences is vital in progressing precise exercise interventions.

#### 3.2.1 Aerobic exercise: a direct and positively ECS activator

Aerobic exercise, such as running, swimming, and cycling, is extensively indicated to be a powerful stimulus for triggering endocannabinoid levels. Its role in ECS regulation is direct and systematic. A classic study in a rat model displayed that aerobic exercise not only drives analgesic effects, but also that this effect can be blocked by CB1 and CB2 receptor antagonists (96). More importantly, a study directly determined that the expression and activation of CB1 receptors were enhanced in the brains of rats after exercise, and the levels of AEA, 2-AG, and related mediators in plasma were significantly increased (96). In human evidence, alterations in circulating endocannabinoid levels after acute or chronic aerobic exercise have been uncovered, tightly linked to enhancements in a scope of subjective emotional states, including decreased feelings of well-being, pleasure, anxiety, depression, and fatigue (76, 97). Together, these findings indicate that aerobic exercise is vital in directly activating the ECS, which in turn modulates mood and perception. Therefore, aerobic exercise plays a central role in directly activating ECS and subsequently regulating mood and perception. In comparison, the regulatory mode of strength training on ECS is more complex and often yields different effects from aerobic exercise (94).

#### 3.2.2 Strength training: indirectly impacts the ECS via neuroplasticity

The modulatory effect of strength training on the ECS is more complex and indirect than aerobic exercise. Recent research cannot consistently display that strength training significantly and directly enhances peripheral endocannabinoid levels as aerobic exercise (95). A study in human skeletal muscle found that acute strength training even decreased plasma levels of 2-AG and while 4 weeks of strength training decreased levels of AEA, PEA, and OEA. The study concludes, Resistance training and endurance training modulate the levels of endocannabinoid ligand and CB1 expression in opposite ways (98). Nevertheless, this cannot imply that strength training is not advantageous for mental health and the ECS. Its advantages may be determined via other mechanisms. Strength training has been reported to drive adaptive modifications in the CNS, that is, enhancements in athletic performance are commonly accompanied by neuroplastic adaptations of the CNS (99). Considering that the ECS is a key regulator of neuroplasticity, strength training may indirectly impact brain function linked to the ECS by promoting the remodeling of neural circuits (79, 100). Moreover, evidence has reported that chronic exercise (including treadmill training) can reverse abnormal elevations of AEA in specific tissues (such as extensor digitorum digits) in certain pathological states, such as high-fat diet-driven obesity rat models, revealing that strength training may normalize or stabilize ECS function under certain conditions (101).

### 3.3 Effects of exercise on the brain’s reward system

**Figure 3:**
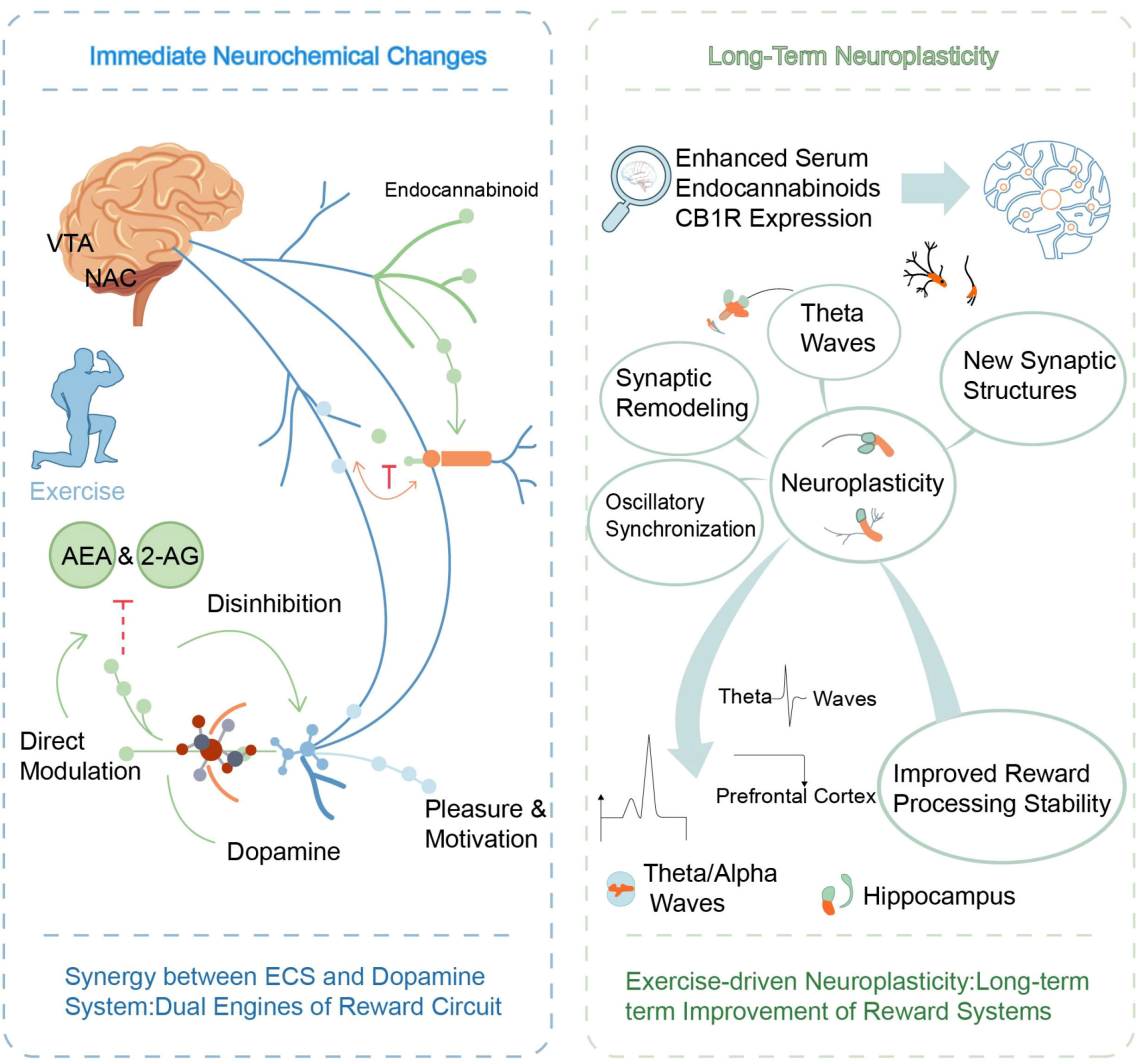
Dual mechanism of exercise regulating brain reward system through the endogenous cannabinoid system. This figure illustrates how exercise acts as a “neural regulatory interface,” linking physical activity to positive emotions via immediate neurochemical shifts and long-term structural adaptation. Left Panel (Immediate Neurochemical Changes): Exercise triggers the release of endocannabinoids (AEA & 2-AG), activating the “Dual Engines of Reward Circuit” in the VTA and NAc through two synergistic pathways: 1) Disinhibition: ECS inhibits upstream inhibitory neurons (indicated by the red “T” bar), indirectly facilitating dopamine release. 2) Direct Modulation: ECS directly stimulates dopamine neurons. These combined actions result in immediate “Pleasure & Motivation.” Right Panel (Long-Term Neuroplasticity): Sustained exercise enhances serum endocannabinoid levels and CB1R expression, driving profound neuroplasticity. Key processes include “Synaptic Remodeling,” the formation of “New Synaptic Structures,” and “Oscillatory Synchronization.” Specifically, the modulation of Theta and Alpha waves in the Hippocampus and Prefrontal Cortex contributes to “Improved Reward Processing Stability,” ultimately securing long-term improvements in the brain’s reward systems. ECS, Endocannabinoid System; AEA, Anandamide; 2-AG, 2-Arachidonoylglycerol; VTA, Ventral Tegmental Area; NAc, Nucleus Accumbens; CB1R, Cannabinoid receptor type 1; DA, Dopamine.

The brain’s reward system is a complex network of neurotransmitters involving dopamine, endocannabinoids, opioid peptides, and more, with its key function of generating pleasure and motivation that drives adaptive behavior (102, 103). Exercise can actively combat the key symptom of depression, namely loss of pleasure, and improve emotional resilience by activating this system. In this process, ECS plays an indispensable neural regulatory interface, playing a role in linking physical activity with positive emotional experiences (104) (50). **Figure 3** shows Dual mechanism of exercise regulating brain reward system through endogenous cannabinoid system.

#### 3.3.1 Synergy between ECS and dopamine system: dual engines of reward circuit

At the heart of the reward system is the mesolimbic dopamine pathway, in which dopamine neurons in the ventral tegmental region project to the nucleus accumbens, releasing dopamine to encode reward prediction and motivational significance (14, 105). The ECS is uniquely positioned to de-influence dopaminergic and serotonergic neurotransmission via the CB1 receptor (13). Exercise-driven elevated levels of AEA and 2-AG can de-inhibit GABAergic interneurons by activating CB1 receptors in reward regions, such as the nucleus accumbens, thereby indirectly promoting dopamine release (106). This disinhibition mechanism is a significant basis for exercise to generate pleasure and reinforce motor behavior. Moreover, as mentioned earlier, CB1 receptors are even directly expressed on neurons that co-release dopamine and glutamate in a subset (21). This offers a more precise cellular basis for eCB to directly modulate reward signal output. The eCB signal improves the function of the dopamine system in both indirect and direct ways, thereby promoting motivation and mood.

#### 3.3.2 Exercise-driven neuroplasticity: long-term improvement of reward systems

The benefits of exercise on the reward system are not limited to immediate neurochemical changes, but are also implied in the persistent structural and functional remodeling of neural circuits, i.e., neuroplasticity (107). Neuroplasticity is fundamental to the brain’s adaptation to environmental challenges, learning, and storage of positive experiences, and it is vital in modulating stress, alleviating depression, and established resilience (12) (107). Substantial evidence reveals that exercise is a powerful means of driving neuroplasticity (107).

This plasticity is determined in part via the mediation of ECS. Physical activity was linked to enhanced serum endocannabinoid concentrations and increased expression of cannabinoid receptor type 1 (CB1R) in the brain, which ultimately caused positive neural effects, including neuroplasticity development (79). This eCB-induced plasticity occurs at numerous levels, from the remodeling of synaptic structures (such as the presynaptic remodeling caused by CB1-iLTD mentioned earlier) to the oscillatory synchronization of large-scale neural circuits. A study of cortical oscillatory activity offer system-level evidence for this (79). Exercise can regulate various types of neural oscillations, and this regulatory effect is closely related to the impact of exercise on neural plasticity. For example, different exercise modes such as resistance training, aerobic training, and physical and mental exercise can improve the function of brain regions such as the prefrontal cortex and hippocampus by regulating specific neural oscillations (such as theta waves and alpha waves), which are closely related to advanced cognition, emotion regulation, and reward evaluation (108). A more coordinated and connected brain network undoubtedly has stronger reward processing capabilities and emotional stability (109).

## 4 Exercise enhances stress resilience and antidepressant mechanisms

**Figure 4:**
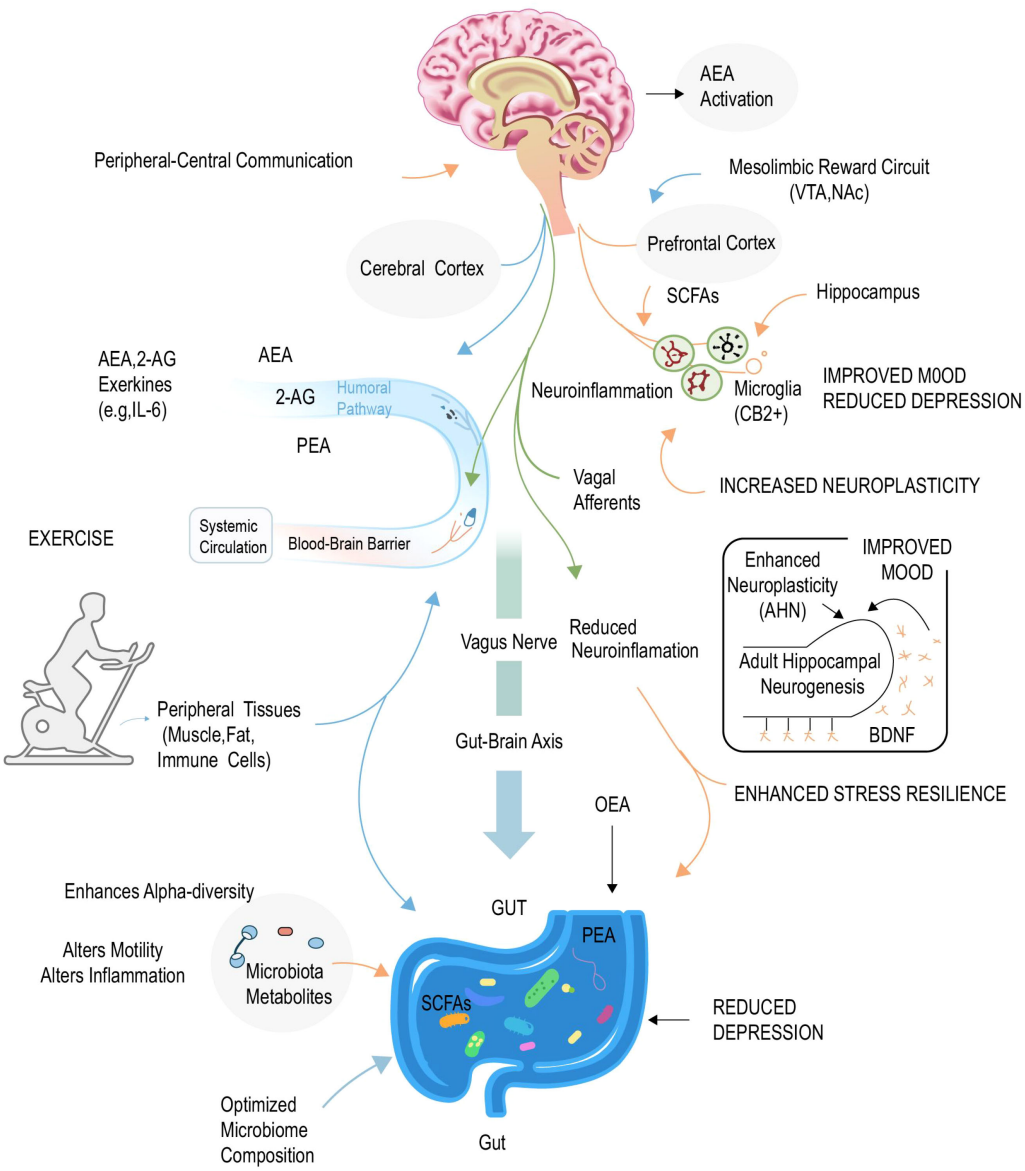
Integrated model of exercise enhancing stress resilience via the microbiota-gut-brain axis and the endocannabinoid system. This figure illustrates the “Peripheral-Central Communication” mechanism by which exercise alleviates depression. The process involves bidirectional signaling between the periphery and the brain: 1) Peripheral & Gut Modulation (Bottom): Exercise optimizes the gut microbiome composition (enhancing $\alpha$-diversity) and stimulates the production of Microbiota Metabolites, particularly Short-Chain Fatty Acids (SCFAs). It also modulates lipid mediators like PEA and OEA in the gut. 2) Transmission Pathways (Middle): These peripheral signals are transmitted to the brain via two primary routes: the Humoral Pathway (where Endocannabinoids like AEA/2-AG and Exerkines like IL-6 cross the Blood-Brain Barrier) and the Neural Pathway (via Vagal Afferents of the Gut-Brain Axis). 3) Central Effects (Top & Right): Upon reaching the brain, these signals target key regions (Prefrontal Cortex, Hippocampus, VTA/NAc). A critical mechanism involves the reduction of Neuroinflammation (mediated by CB2+ Microglia) and the enhancement of Neuroplasticity. As shown in the inset, this leads to increased Brain-Derived Neurotrophic Factor (BDNF) levels and Adult Hippocampal Neurogenesis (AHN), ultimately resulting in improved mood and enhanced stress resilience. ECS, Endocannabinoid System; AEA, Anandamide; 2-AG, 2-Arachidonoylglycerol; PEA, Palmitoylethanolamide; OEA, Oleoylethanolamide; SCFAs, Short-Chain Fatty Acids; VTA, Ventral Tegmental Area; NAc, Nucleus Accumbens; AHN, Adult Hippocampal Neurogenesis; BDNF, Brain-Derived Neurotrophic Factor; CB2, Cannabinoid receptor type 2.

The occurrence and development of depression are not only related to the failure of the internal reward system, but also closely related to an individual’s ability to adapt and recover from external environmental challenges, especially chronic or significant stress (110). This ability, namely resilience to pressure, is considered a key psychological barrier against emotional disorders, and its biological basis is closely related to ECS mediated steady-state regulation and neural plasticity (110). **Figure 4**. Dual pathway integrated model of exercise enhancing compressive toughness through endogenous cannabinoid system.

### 4.1 The correlation between stress resilience and depression

Stress resilience is an individual’s dynamic ability to positively cope with and sustain adaptability in the face of psychological and physical stress. This ability is not only implied in immediate emotional stability under stress exposure, but also in the long-term potential to quickly regain homeostasis from stress (111). Stress resilience is closely related to the occurrence, development, and recovery of depression. Low stress resilience is an important risk factor for depression, while high stress resilience is a key psychological barrier to resist emotional disorders (111).

#### 4.1.1 The effect of stress resilience on the occurrence and recovery of depression

A large number of empirical evidence has determined that stress resilience levels are poorly linked to the severity of depressive symptoms. In the context of the global stress of the COVID-19 pandemic, research has uncovered that higher mental resilience is linked to lower levels of anxiety and depression, as well as enhanced ability to cope with the constant stress of daily life (112). A systematic review has further indicated the prevalence of this correlation, pointing out that resilience was inversely linked to depression and anxiety during the COVID-19 pandemic (113). Conversely, low-stress resilience may cause individuals to overreact to stress from poor events, which can boost or worsen depression. A study of Chinese adolescents found, The association between poor life events and depressive symptoms is partly induced by resilience (114).This reveals that stress resilience plays a key inducing role between poor life events and depressive symptoms – poor life events increase depression risk by decreasing an individual’s resilience resources. Therefore, increasing individual stress resilience is not only considered a significant approach for depression prevention, but also a key goal in the treatment process.

#### 4.1.2 The role of ECS in enhancing stress resilience

The ECS is vital in modulating mood, stress response, and neuroplasticity, and is a significant biological basis for stress resilience (115). The ECS helps individuals sustain homeostatic balance in stress coping by modulating neurotransmitter release and decreasing the secretion of stress hormones, such as cortisol (17).

The regulatory role of ECS in stress response is notably critical. The evidence reviewed here reveals that endocannabinoid signaling is involved in activating and terminating the hypothalamic-pituitary-adrenal axis response to acute and recurrent stress (16). This bidirectional modification of the HPA axis makes the ECS an ideal pressure buffer system – both determining the necessary stress response and avoiding over-response or prolongation (71). Literature has reported that there is a direct association between the normal function of the ECS and stress resilience. Increasing ECS activity significantly promotes the ability to cope with stress, and the ECS (ECS) modulates our responsiveness to stress, including neuroendocrine and behavioral stress, via the corticolobar system and the HPA axis (116). It is particularly noteworthy that the crosstalk between ECS and gut microbiota plays an important role in pressure recovery. The gut microbiota and endogenous cannabinoid system are both believed to be related to stress recovery ability (117).At the molecular level, the 2-AG signaling pathway plays a special role in enhancing stress resilience, emphasizing the protective role of 2-AG in stress response termination and stress resistance (57). This protective effect is obtained via swift synaptic regulation – in the stress state, the swift synthesis of 2-AG and retrograde inhibition of glutamate over elease via CB1 receptors, thereby restricting the intensity and duration of the stress response.

### 4.2 process of exercise to increase stress resilience

#### 4.2.1 Modulation of exercise via the ECS

Exercise is a powerful physiological activator of the ECS. Cross-species study has determined that exercise-driven eCB signaling is significantly increased in humans and dogs after high-intensity endurance running (77). This finding suggests the evolutionary conservation of the exercise-activated ECS. More systematic evidence comes from a meta-analysis that suggested a coherent-enhancing effect of acute exercise on eCBs: the meta-analysis displayed significant increases in AEA and 2-AG after acute exercise in various patterns species and with or without pre-existing health conditions. The study further reports that the ECS plays a key role in sustaining homeostasis, and that chronic stress may hinder eCB signaling, so exercise as a behavioral intervention targeting the ECS may be a promising treatment for the prevention and treatment of stress-associated disorders (118). Exercise-driven enhanced levels of AEA and 2-AG exert their emotion-regulating and neuroprotective effects by activating extensively distributed CB1 receptors in the brain (77). This is supported by studies showing that pharmacological potentiation of the ECS after severe stress can prevent the development of depressive- and PTSD-like behaviors, and crucially, normalize stress-induced reductions in BDNF levels within key brain regions such as the hippocampus and prefrontal cortex (119). This mechanism promotes fine management of emotion modulation and increases the ability to cope with stress. via these biological mechanisms, exercise can systematically enhance the brain’s tolerance to stress and its ability to recover from stress, thereby significantly decreasing the risk of depression.

#### 4.2.2 Effects of exercise on key regions, such as the cerebral cortex and hippocampus

The cerebral cortex and hippocampus are key nodes in modulating mood and stress, and exercise can increase the neuroplasticity of these areas and improve the improvement of their structure and function, which is the neuroanatomical basis for the formation of stress resilience (120).The hippocampus, as a component of the limbic system, is a central brain region for emotion modulation and memory formation, and its function is modulated by adult hippocampal neurogenesis (AHN) and is considered to be a key brain region that modulates stress response (121). AHN represents a superior form of neuroplasticity that, via its computational influence, is vital in comprising adaptation to environmental demands (122). AHN appears to favor hippocampal computation toward increased connectivity coding and pattern separation, promoting contextual discrimination and cognitive flexibility, and these effects enable downstream brain regions to receive more precise and contextualized information, allowing different dimensions of the stress response to be more appropriately matched to specific contexts (123). Exercise is one of the most proactive non pharmacological interventions to improve hippocampal neurogenesis. Exercise is widely recognized for its numerous neuroprotective and cognitive benefits, particularly in memory and learning related processes (15, 124). One potential mechanism of association is motor induced hippocampal neurogenesis, which refers to the process of generating and integrating new neurons into the hippocampus circuit. This exercise induced neurogenesis is closely related to brain-derived neurotrophic factor (BDNF), which is itself regulated by ECS and forms a positive cycle that promotes plasticity (125). Similarly, there is evidence to suggest that moderate levels of exercise can promote neurogenesis in the adult brain. From the perspective of neurogenesis, we have summarized the evidence of the impact of exercise on hippocampal neurogenesis.

BDNF is a key molecule mediating neuroplasticity, neuronal survival, and growth, and its functional deficiency is closely related to the pathophysiology of depression. Clinical studies have directly confirmed that exercise intervention can effectively elevate BDNF levels in patients with depression. A randomized controlled trial targeting inpatients with depression found that after receiving a 6-week structured, supervised exercise intervention, the serum BDNF levels in the exercise group significantly increased, while those in the conventional treatment group slightly decreased, with a significant interaction effect between the two groups (p=0.030) (126). This result indicates that supplementing standard treatment with exercise can exert additional positive effects on BDNF levels in patients with depression. Earlier landmark studies have established a causal chain of “exercise → increased hippocampal volume → elevated BDNF levels → improved memory” in healthy elderly individuals, demonstrating that aerobic exercise training can increase the volume of the anterior hippocampus by 2% and reverse age-related atrophy, and these increases in hippocampal volume are significantly correlated with elevated serum BDNF levels (127). These findings provide crucial support for the hypothesis that exercise promotes brain plasticity in patients with depression by upregulating BDNF. Although animal models and studies in healthy individuals strongly suggest that exercise promotes hippocampal neurogenesis and volume increase, clinical evidence in patients with depression presents a more complex and contradictory picture. Studies have found a positive correlation between changes in hippocampal volume after exercise intervention and improvements in clinical symptoms. For example, although one study did not observe significant differences in total hippocampal volume between the exercise and control groups, *post-hoc* analysis revealed a positive correlation between changes in hippocampal volume and improvements in depressive symptoms (Rho=0.30; p=0.03) (128). This suggests that the plasticity of hippocampal structures may be involved in mediating the emotional benefits of exercise. However, not all studies have yielded positive results. The primary endpoint analysis of the same study did not observe a significant increase in overall hippocampal volume in outpatient patients with mild to moderate depression after exercise intervention (128). A systematic review and multi-level meta-analysis up to 2025 further summarized the current state of the field: in animal depression models, exercise has a significant large effect on hippocampal volume (g = 0.90); however, in studies of human patients with depression, the average effect size of exercise is very small and non-significant (g = 0.05) (129). These contradictory findings may stem from various factors. In clinical practice, the “dose” of intervention (such as frequency, intensity, duration) may not be sufficient to induce significant structural changes observed in healthy elderly populations; patient factors such as severity of depression, duration of illness, pharmacological treatment, and exercise adherence (with some studies indicating an average participation rate of only once per week) (128) may also influence the results as confounding variables. Additionally, different subregions of the hippocampus (such as the dentate gyrus.

### 4.3 Connective pathways between peripheral and central endogenous cannabinoid systems

The regulation of ECS by exercise is not limited to the central nervous system, but rather represents an integrated process involving multiple organ systems throughout the body. Peripheral tissues (such as fat, muscle, immune cells, and the gut) also undergo significant ECS changes under the stimulation of exercise. These peripheral signals dynamically and bidirectionally communicate with the central ECS through multiple parallel physiological pathways, collectively coordinating the body’s stress adaptation and emotional homeostasis. The main connecting pathways include humoral transport, neural conduction, and immune regulation.

Endogenous cannabinoids, such as AEA and 2-AG, are lipid-soluble signaling molecules that can be synthesized and released into the blood circulation under stimuli such as exercise (104). Although their specific transport mechanisms across the synapse in the brain are still under in-depth investigation, these molecules can cross the blood-brain barrier through passive diffusion or specific transport mechanisms, directly affecting the ECS activity in the central nervous system (130). Meta-analyses have confirmed that acute exercise consistently elevates the levels of circulating endogenous cannabinoids, including AEA and 2-AG, and this elevation is associated with the mood-enhancing effects post-exercise (118). Clinical studies have also observed that a single session of acute aerobic exercise can elevate the levels of AEA and related lipid mediators in the plasma of cancer patients and healthy controls, and this is associated with the subjective experience of “runner’s high” (131). This suggests that endogenous cannabinoids in the circulation may act as “exercise messengers”, directly transmitting peripheral physiological signals of exercise to the brain, translating them into immediate mood-improving effects, and constituting the most direct peripheral-central connection pathway.

In addition to the humoral pathway, peripheral ECS signals can also affect the brain through a rapid neural pathway. The core of this pathway is the vagus nerve, especially its enteric afferent fibers. Activation of ECS in peripheral tissues (especially the intestine) or eCBome substances (such as OEA and PEA) produced by gut microbiota metabolism can act on various receptors (such as CB1, TRPV1, PPAR-α) expressed on the vagal nerve endings in the intestine. These activated neural signals first transmit to the nucleus tractus solitarius (NTS) of the brainstem for relay processing, and then project to brain regions closely related to emotion, stress, and reward, such as the hypothalamus, amygdala, and ventral tegmental area (VTA). Studies have confirmed that the vagal afferent pathway is a key channel mediating the influence of gut microbiota on the mesolimbic dopamine reward system (132). Therefore, this pathway provides a high-speed neural communication foundation for the “gut-brain axis”, explaining how intestinal ECS status and microbiota metabolites can rapidly and precisely regulate central emotional and motivational behaviors.

Exercise provides another important pathway connecting peripheral and central ECS by regulating systemic low-grade inflammation. Exercise can trigger the release of a series of cytokines known as “exerkines,” such as elevating interleukin-6 (IL-6) levels in the short term and reducing tumor necrosis factor-α (TNF-α) and C-reactive protein (CRP) levels, thereby inducing a systemic anti-inflammatory shift (133). Peripheral immune cells widely express CB2 receptors, whose functions are regulated by the ECS. Simultaneously, these peripheral inflammatory signals can penetrate the blood-brain barrier or activate cerebral vascular endothelium, affecting microglia within the central nervous system. Microglia are inherent immune cells of the brain, and their activation and phenotypic conversion are critically regulated by CB2 receptors, thereby balancing the central pro-inflammatory and anti-inflammatory responses (134). Therefore, the peripheral anti-inflammatory effects induced by exercise can interact with central ECS functions (especially CB2 receptor signaling) through a common pathway of regulating neuroinflammation, linking the peripheral health benefits of exercise with central mood improvement and neuroprotective effects (18).

In summary, the regulation of ECS by exercise is a systemic and multi-pathway coordinated process. The activation of peripheral ECS forms a dynamic and bidirectional communication network with central ECS through three main pathways: humoral transport, vagal afferent, and immune regulation. It is particularly noteworthy that gut microbiota and its metabolites ingeniously integrate and utilize these pathways (especially the vagal pathway and humoral pathway), thereby becoming a key hub and functional amplifier connecting exercise, peripheral ECS, and central reward/emotional circuits. This multi-level, multi-pathway integration mechanism ensures that exercise, as a behavioral intervention, can systematically restore the homeostasis of the endocannabinoid system, thereby effectively enhance an individual’s stress resilience and exerting its antidepressant effect.

### 4.4 Gut microbiota: the cross-disciplinary bridge connecting exercise, ECS, and the brain reward system

Gut microbiota plays an indispensable role in exercise-mediated mood improvement and antidepressant effects. As a “cross-border bridge” connecting peripheral physiological activity and central nervous system function, it interacts closely with the endocannabinoid system, jointly constituting another key pathway through which exercise exerts its benefits.

Regular exercise serves as a potent non-pharmacological intervention for shaping a healthy gut microbiota. Evidence from systematic reviews and meta-analyses indicates that exercise interventions can significantly enhance the alpha diversity of gut microbiota in the adult population, as exemplified by the increase in the Shannon index (135). This regulatory effect is bidirectional: moderate exercise promotes immune health, enhances the integrity of the intestinal barrier, and increases the production of beneficial metabolites such as short-chain fatty acids (SCFAs); whereas prolonged high-intensity exercise may temporarily disrupt intestinal balance (136). Exercise optimizes the composition and function of the microbiota through multiple pathways, including alterations in intestinal motility, local oxygen tension, bile acid secretion profile, and systemic inflammatory status, laying the foundation for the subsequent production of neuroactive metabolites (137).

Gut microbiota, especially beneficial bacteria such as lactobacilli and bifidobacteria, can metabolize dietary components to produce a series of lipid signaling molecules. Among them, compounds with structures similar to classical endocannabinoids, such as N-oleoyl ethanolamide (OEA) and N-palmitoyl ethanolamide (PEA), have garnered significant attention (138). These substances, along with AEA, 2-AG, and others, belong to a broader signaling network known as the endocannabinoidome (eCBome), which is an important extension of the ECS function (139). Exercise can regulate the microbiota, altering the production of these eCBome lipid mediators. The elevated levels of PEA, OEA, and other compounds in peripheral circulation after exercise are partly attributed to exercise-driven metabolic changes in the microbiota (137). These metabolites derived from the microbiota serve as key messengers linking the gut environment with the functional status of the ECS throughout the body, including the brain.

These mycotoxin-derived cannabinoid-like metabolites primarily affect the central reward system through two pathways: (1) The vagal pathway: Substances such as OEA and PEA produced in the gut can activate specific receptors (such as PPAR-α and TRPV1) expressed on the afferent terminals of the gut’s vagal nerve (140). After being relayed through the nucleus tractus solitarius (NTS), the signal can further project to the ventral tegmental area (VTA) of the midbrain, ultimately regulating dopamine release in the nucleus accumbens (NAc). Studies have confirmed that the vagal afferent pathway mediates the regulatory effect of the gut microbiota on the mesolimbic dopamine system (132). (2) The humoral pathway: Some metabolites of the gut microbiota can enter the systemic circulation through the intestinal barrier (141). They either cross the blood-brain barrier or act on the nerve endings surrounding the cerebral blood vessels, indirectly regulating the activity of the central nervous system’s endocrine-immune system (ECS) and neuroinflammatory states, thereby affecting mood and reward processing (142).

In summary, “exercise → gut microbiota regulation → eCBome metabolite production → activation of the vagal/humoral pathway → central reward circuitry (dopamine release)” constitutes a complete “gut-brain axis”. This peripheral signaling pathway parallels and synergizes with the pathway through which exercise directly elevates central AEA/2-AG and activates CB1 receptors, jointly elucidating how exercise enhances resilience, combats depression, and reshapes reward experiences by reshaping endocannabinoid signaling in a systematic manner. This provides a more integrated and profound biological perspective for understanding the emotional benefits of exercise.

## 5 The potential of exercise and the ECS in the treatment of depression

### 5.1 Benefits of exercise as a non-pharmacological intervention

#### 5.1.1 Safety and sustainability compared to drug treatment

Although antidepressants are currently the mainstream treatment, their long-term limitations cannot be ignored. A key issue is that antidepressants can trigger dependence and withdrawal reactions, which may be one of the reasons for unnecessary long-term prescriptions (3). Moreover, tolerance during long-term antidepressant therapy may cause worse outcomes in several patients with depression, that is, the phenomenon of loss of effectiveness in long-term treatment, also poses a clinical challenge (143).

Conversely, exercise interventions display significant safety and sustainability benefits. Evidence suggests that exercise typically provides benefits comparable or even better than drug intervention, without any side effects or economic burden (144). What’s more, the benefits of exercise expand beyond mental health to systemic physiological systems: enhanced physical activity and exercise are linked to a decreased risk of chronic disease (144). Most physiological systems in the body derive positive advantages from PA and exercise via disease prevention and secondary disease prevention/treatment (145). This numerous health advantages make exercise a highly cost-effective and sustainable health control strategy.

#### 5.1.2 Comprehensive improvement of individual quality of life and mental health

The therapeutic value of exercise lies not only in the relief of symptoms, but also in its positive promotion of the individual’s overall quality of life and psychological function (146, 147). A meta-analysis of patients with chronic brain disease showed that exercise is an positively and safe add-on therapeutic intervention with a moderate effect on quality of life and a significant effect on mood (148). Of particular note is the promoting effect of exercise on mental toughness. A systematic review and meta-analysis targeting young students found a significant positive correlation between physical activity and psychological resilience, clearly indicating that physical activity has a positive impact on psychological resilience (149). In patients with depression, exercise has a notably significant effect on promoting quality of life. Exercise significantly promoted the physical and mental domains as well as overall quality of life, while the control group lacked enhancement, reinforcing the positive role of exercise as a treatment for depression and promoting quality of life (146).

### 5.2 Prospects for clinical application

ECS-based exercise interventions open up a new aspect on the treatment of depression. By incorporating the modulatory effects of exercise on ECS into the therapeutic framework, it is expected that more targeted, mechanism-induced, and individualized non-pharmacological intervention approaches will be developed in the future, thereby strengthening the recent treatment landscape for depression (118).

The key to translating the regulatory process of the ECS into clinical practice lies in developing precise exercise prescriptions. Clinical doctors should design personalized exercise plans based on the severity of depression, physical condition, exercise history, and personal preferences of patients, with the core principle of optimizing ECS activation (150, 151). Among them, precise control of exercise intensity is crucial (150). Research has shown that eCB signals are indeed linked to exercise intensity, and significant changes in circulating eCB levels were only observed at moderate intensity (neither extremely high nor low intensity exercise significantly changed circulating eCB levels). This discovery directly guides clinical practice: to maximize the emotional improvement effect mediated by ECS, moderate intensity aerobic exercise (such as brisk walking, jogging, cycling, reaching 60% to 75% of maximum heart rate) should be prioritized (152, 153). This is consistent with the research findings in the field of emotion modulation, that is, the effect of aerobic exercise on emotion modulation is influenced by the intensity and duration of exercise.

### 5.3 individualized exercise therapy

A key consensus in clinical translation of using exercise as an intervention for depression is that a ‘one size fits all’ approach is far from optimal. There are significant differences in individual health status, severity of depression, exercise tolerance, and physiological responses. Therefore, developing personalized exercise plans is crucial for maximizing treatment effectiveness, determining safety, and promoting long-term compliance.

#### 5.3.1 Individualized selection of sports types

The choice of exercise type should be according to the patient’s initial functional status and personal preference for stepped planning. For people with early depression, older patients, or those with negative physical condition, low-intensity, easy-to-perform aerobic exercise is an ideal place to start (153). A systematic review and meta-analysis of 75 randomized controlled trials (RCTs) concluded that different forms of walking can effectively alleviate symptoms of depression and anxiety. Specifically, it was pointed out that patients with depression may benefit more, and this benefit is not significantly affected by walking frequency, duration, location, or specific form, providing high flexibility for clinical practice (154). As the patient’s physical and mental condition improves, the treatment plan should be gradually adjusted and promoted. For patients with long-term or treatment-resistant depression, more structured moderate-intensity aerobic exercise or combined with strength training can be described on the basis of low-intensity aerobic exercise to synergistically promote physical and psychological adaptability (155). An RCT targeting outpatient depression patients found that combining 12 weeks of aerobic exercise with routine psychiatric treatment significantly decreased Hamilton Depression Scale scores and improved sleep quality, providing strong evidence that aerobic exercise may become an effective adjunctive treatment for depression (156).This combined approach seeks to regulate ECS function, increase neuroplasticity, and promote physical health via various types of exercise.

#### 5.3.2 Correlation between exercise intensity and effect

Precise control of exercise intensity is the key to individualized prescription. Literature has reported that exercise has an intensity-dependent effect on ECS activation (157). eCB signaling is intensity-dependent, and significant modifications in circulating eCB were uncovered only after moderate intensity (very high and very low intensity exercises cannot significantly alter circulating eCB levels) (10). This finding offers a potential molecular biological explanation for the optimal mood-triggering effect of moderate-intensity exercise. However, high-intensity exercise is not suitable for all patients. Although it can more effectively activate the endogenous cannabinoid system and reward system, it may also increase the burden on the body (157). Subjects exposed to high-level physical activity can impede cardiovascular health, and overloading training can exacerbate strong oxidative stress, damaging proteins, glucose, lipids, and nucleic acids in cells (157). Therefore, clinicians must carefully alter the intensity of exercise based on the patient’s physical condition, exercise experience and tolerance, starting with low intensity and gradually and slowly increasing to prevent excessive physical and psychological stress, which is notably vital in patients with depression.

#### 5.3.3 Polymorphisms in ECS-related genes and genes related to neuroplasticity

##### 5.3.3.1 ECS gene polymorphism

The function of ECS is influenced by genetic variations in its constituent genes, which may regulate the baseline response of individuals to stress, reward stimuli, and potential behavioral interventions such as exercise.

FAAH is a key enzyme that degrades AEA. The human FAAH gene exhibits a functional polymorphism (C385A, rs324420), where the A allele is associated with reduced FAAH enzyme activity and elevated AEA levels. Studies indicate that this polymorphism may be related to emotional regulation and depression risk. A large-scale study focusing on adolescents during the pubertal transition found that individuals carrying the AA genotype exhibited significantly lower depressive symptoms at both time points compared to those with AC and CC genotypes (158). However, its relationship with emotional reactivity may be complex and bidirectional. Another study revealed that compared to C/C genotype carriers, A allele carriers exhibited enhanced startle reflex potentials (indicating increased emotional reactivity) to unpleasant picture stimuli, while their startle reflex inhibition to pleasant pictures was reduced (indicating decreased reactivity) (159). Furthermore, this polymorphism has also been linked to the neural basis of fear extinction memory, with A allele carriers (AC genotype) showing stronger neural activation in key brain regions such as the anterior cingulate gyrus and insula during extinction recall tasks, accompanied by lower anxiety state scores, suggesting they may possess more successful extinction memory abilities (160). These findings suggest that the FAAH genotype may potentially modulate the emotional benefits of behavioral interventions such as exercise by regulating the availability of AEA, thereby affecting an individual’s processing of emotional stimuli, adaptability to stress learning, and susceptibility to anxiety and depression.

The polymorphism of the CNR1 gene, which encodes the CB1 receptor, is also associated with neuropsychiatric phenotypes. For instance, the single nucleotide polymorphism rs2023239 is related to the binding affinity of CB1 receptors in the brain, subjective reward sensation after alcohol intake, and reward-related brain activation (161). Studies indicate that this risk allele (C) interacts with stressful life events in childhood and adulthood, jointly predicting problematic alcohol use. In terms of major depressive disorder (MDD), research has found that CNR1 SNPs (such as rs806367 and rs6454674) and their specific haplotypes are associated with increased susceptibility to MDD and resistance to antidepressant treatment (162). Furthermore, studies suggest that ECS signaling regulators (such as CNR1 genotype) differentially modulate the brain’s neural response to reward cues (including drugs and natural rewards) in conjunction with stress factors (163). This implies that an individual’s CNR1 genotype may indirectly affect their ability to benefit from exercise (a natural rewarding activity) by influencing their neural sensitivity to stress and reward.

##### 5.3.3.2 Neuroplasticity-related gene polymorphism: taking BDNF as an example

Brain-derived neurotrophic factor (BDNF) is a core mediator of exercise-induced neuroplasticity. The human BDNF gene harbors a common nonsynonymous polymorphism (Val66Met, rs6265), where the Met allele impairs activity-dependent BDNF secretion and is associated with a range of neuropsychiatric phenotypes (164). This polymorphism serves as a significant genetic factor influencing hippocampal structure and function, stress response, and depression risk.

Research has confirmed that compared to individuals with Val/Val homozygotes, patients carrying the Met allele and healthy controls exhibit significantly reduced hippocampal volumes (165). In cellular and animal models, Met-BDNF exhibits impaired activity-dependent secretion (166). Importantly, this polymorphism directly affects exercise-induced neuroplasticity and behavioral improvement. In gene knockout mice carrying the human BDNF Val66Met polymorphism, exercise training significantly reduced the latency period in the novel environment-induced food intake suppression test and the immobility time in the forced swim test in wild-type (BDNFVal/Val) mice, but had no such effect on BDNFMet/Met homozygous mice (167). Exercise also increased hippocampal neurogenesis and enhanced the expression of BDNF and its related genes (such as FNDC5) in the hippocampus and muscles in wild-type mice, but these beneficial changes were attenuated or completely absent in BDNFMet/Met mice (167). This key evidence directly indicates that the BDNF Val66Met polymorphism is a key genetic factor regulating the behavioral and neurobiological benefits individuals obtain from exercise.

### 5.4 Challenges and critical perspectives in clinical translation

#### 5.4.1 Heterogeneity of clinical efficacy and methodological challenges

Although multiple meta-analyses have confirmed a moderate effect size of exercise on depressive symptoms (168, 169), the results of randomized controlled trials (RCTs) exhibit significant heterogeneity. Some high-quality studies have failed to demonstrate the significant advantage of exercise intervention compared to active control groups (such as health education, stretching exercises). For instance, a systematic review pointed out that when compared to active controls, exercise intensity did not show consistent significant differences in improving depression scores (170). Another RCT targeting outpatient depression patients also found that although the depression scores decreased in the exercise group, significant improvements were observed in both groups compared to the control group receiving conventional care, and the advantage of the exercise group was not prominent (156). This inconsistency mainly stems from the complexity of methodology. The vast differences in exercise type, intensity, frequency, and duration make direct comparisons between studies difficult. There is no consensus on the “optimal exercise prescription” for depression and ECS activation; moreover, unlike drug trials, exercise interventions cannot achieve double-blindness for participants or therapists, and strong expectation and attention effects may overestimate the specific efficacy of exercise itself; patients’ depression subtypes, severity, comorbidities, background of drug treatment, as well as baseline physical fitness and exercise habits, all significantly modulate the response to exercise therapy. Currently, most studies have not conducted sufficient pre-stratification analysis for these subgroups.

#### 5.4.2 Evidence contradictions and measurement limitations of ECS as an intermediary biomarker

Positioning ECS as the core molecular pathway through which exercise produces emotional benefits still requires addressing some inconsistent experimental findings and measurement challenges:

There is uncertainty regarding the correlation between the periphery and the central nervous system. Most human studies infer ECS activity by measuring the levels of AEA and 2-AG in circulating blood (171). However, whether peripheral eCBs concentrations can accurately and dynamically reflect ECS signaling activity in key areas of the limbic system (such as the nucleus accumbens and amygdala) remains a fundamental question that has not been fully resolved. Studies have shown that peripheral AEA levels are negatively correlated with emotional regulation ability, but not significantly associated with specific psychiatric disorder diagnoses or state anxiety and depression scores (172), suggesting that the regulation of the blood-brain barrier makes the correspondence between the periphery and the central nervous system complex and nonlinear.

Furthermore, there is inconsistency in the exercise response. Although studies support that moderate-intensity aerobic exercise can increase peripheral AEA and 2-AG levels (81, 104), other studies have found that the patterns of changes in these molecules after resistance training are different or even opposite. Acute resistance exercise can lead to a significant decrease in peripheral AEA levels (95), and long-term resistance training can also reduce the plasma levels of AEA and 2-AG (98). Additionally, the strength of the correlation between mood improvement and changes in eCBs levels varies greatly across different studies (173), suggesting the presence of underrecognized moderating variables (such as individual genetic background, sampling time points). The complexity of ECS involvement is further exemplified by findings that the spontaneous activity of CB2 receptors is specifically required for the antidepressant-like and pro-neurogenic effects, but not the anxiolytic effects, of a conventional SSRI antidepressant in stressed mice (84). This underscores that the ECS’s role is not monolithic but involves receptor- and behavioral endpoint-specific mechanisms, complicating its straightforward adoption as a biomarker.

Direct clinical evidence for the neuroplasticity hypothesis needs to be strengthened. Although animal models clearly demonstrate that exercise promotes BDNF release and hippocampal neurogenesis through the endocannabinoid system (ECS) (174), and in healthy individuals, a single session of moderate-intensity exercise leads to memory improvement associated with simultaneous increases in anandamide (AEA) and BDNF levels (175), longitudinal clinical studies directly verifying the complete causal chain of “exercise → ECS activation → BDNF/hippocampal structural changes → symptom relief” in patients with major depressive disorder (MDD) are still relatively scarce. Some clinical studies show that exercise can increase serum BDNF levels and is associated with symptom improvement (176), but direct human evidence indicating that changes in the ECS are an indispensable link in this chain still needs to be accumulated, and the effectiveness of BDNF as a biomarker for the efficacy of exercise remains controversial (177).

#### 5.4.3 Toward precision medicine: genetic susceptibility and individual differences

The significant interindividual variability in response suggests that future clinical translation must move beyond a one-size-fits-all approach and toward precision medicine. Genetic factors provide a crucial perspective in this regard.

The function of the endocannabinoid system is influenced by genetic variations. For instance, functional polymorphisms (such as C385A) in the fatty acid amide hydrolase (FAAH) gene can affect the degradation rate of anandamide (AEA), which is associated with anxiety, depressive traits, and neuroendocrine responses to stress. Studies have shown that the A allele interacts with childhood adversity and may increase the risk of anxiety and depression (178), and participates in emotional processing by affecting prefrontal-amygdala function (179). Polymorphisms in the cannabinoid receptor 1 (CNR1) gene, such as rs2023239, have also been studied in relation to reward processing, depression risk, and response to antidepressants (162). Studies have found that the CNR1 rs1049353 G allele is associated with an increased risk of resistance to antidepressant treatment and a reduced subcortical brain response to social reward stimuli, such as happy faces (180). These variations are also likely to modulate an individual’s sensitivity to exercise-induced ECS activation and subsequent emotional benefits.

The Val66Met polymorphism in the brain-derived neurotrophic factor (BDNF) gene is a key regulator of neuroplasticity and stress response. The Met allele impairs activity-dependent BDNF secretion, which is associated with hippocampal dysfunction and memory decline (166), and is considered a susceptibility modifier for various psychiatric disorders (181). Exploring whether this polymorphism affects exercise-induced neuroplasticity and antidepressant efficacy is an important research direction for individualized exercise prescription.

## 6 Conclusions and prospects

### 6.1 Summary

The central thesis of this review is that exercise fundamentally establishes the brain’s stress resilience by functioning as an endogenous activator of the ECS and reversing depression-associated ECS hypofunction. It is worth noting that at the mechanistic level, the ECS is the key system for sustaining neurohomeostasis and its dysfunction (manifested as endocannabinoid deficiency) is a significant pathological basis for the failure of the reward circuit, hyper stress response and neuroinflammation in depression. Exercise, notably moderate-intensity aerobic exercise, can significantly enhance AEA and 2-AG levels, and not only generate immediate mood boost and analgesic effects (such as explaining the runner’s high) by activating extensively distributed CB1 receptors, but also obtain long-term structural optimal programming of mood and stress circuits by driving protein synthesis and ubiquitination-dependent presynaptic remodeling, promoting hippocampal neurogenesis, and regulating neuroimmunity. At the functional level, the above molecular and cellular events eventually converge into a significant increase of behavioral stress resilience. A motion-strengthened ECS system can more precisely initiate and terminate the stress response on the HPA axis, improve the fading of fear memories, and sustain emotional stability, thereby established a strong neurobiological foundation for resisting depression recurrence and coping with life’s challenges. At the intervention level, ECS-regulated individualized exercise therapy has reported unique benefits over traditional drugs, including high safety, systemic health benefits, and endogenous rewards and effective reinforcement induced by the ECS itself, thereby promoting treatment adherence.

### 6.2 Clinical significance

Advances in this range have brought a revolutionary aspect to the prevention and treatment paradigm of depression. First, it offers a mechanism-induced solution that goes beyond the empirical observation that exercise is beneficial and offers a compelling scientific basis for combining exercise prescription into clinical guidelines, elevating it from an adjunctive to a mechanistically defined independent or synergistic treatment strategy. Moreover, this opens up a new path of non-pharmacological treatment, and exercise interventions targeting ECS offer a positively alternative for patients with drug intolerance, poor effectiveness, or contraindications, notably for avoiding depression recurrence and established psychological resilience for all. Also, this has improved the practice of precision psychiatry, and recognizing the causal link between exercise-ECS-depression is a key step in obtaining individualized medicine. In the future, clinical practice can tailor exercise prescriptions based on the patient’s ECS function phenotype, genetic backdrop and lifestyle to obtain more accurate intervention.

### 6.3 Future prospects

Despite the increasingly clear chain of evidence, translating this cutting-edge knowledge into routine clinical practice still requires exploration in the following key directions. There is an urgent need to investigate objective indicators that can predict exercise effectiveness, such as baseline plasma eCBs levels, imaging indicators of CB1 receptor availability in the brain, or related gene polymorphisms, to obtain true stratification and precision medicine. Utilizing advanced tools, such as multimodal neuroimaging, chemogenetics and optogenetics, the dynamic process of motor regulation ECS impacting the functional network of the whole brain is particularly depicted *in vivo* and spatiotemporal, and the full signaling pathway from peripheral somatic movement to central emotional improvement is elucidated. It is necessary to systematically deconstruct the elements (mode, intensity, frequency, duration) of exercise prescription via large-scale and RCTs, build dose-effect correlations for various depression subgroups and various disease stages, and form standardized operating guidelines. The synergistic effects of exercise and multimodal interventions, such as medication, psychotherapy, and physical therapy are studied, notably whether they can accelerate the onset of traditional antidepressants, increase effectiveness, or decrease side effects, so as to construct an combined treatment plan. Further suggest the crosstalk between ECS and gut microbiome, metabolome and other systems, determine the overall biological basis of exercise advantages from the aspect of systemic physiological networks, and progress a remote controlling and guidance system combined with mobile health technology to improve the large-scale translation of research results.
