# Correction: Bridging reward and resilience: the endocannabinoid system as a unifying mechanism in exercise-induced protection against major depressive disorder

**DOI:** 10.3389/fpsyt.2026.1835045

**Published:** 2026-06-11

**Authors:** Guanmin Zhang, Qiuju Hu, Haiyang Zou

**Affiliations:** 1 Department of Physical Education, Gangneung-Wonju National University, Gangneung, Republic of Korea; 2 Department of Physical Education, Henan Technical Institute, Zhengzhou, , Henan, China

**Keywords:** ECS, major depressive disorder, motion, neuroplasticity, non-pharmacological interventions, reward system, stress resilience

In the published article, several citations were embedded in the text as full references and were missing from the reference list. The following references have been added to Reference list and the in-text citations reformatted:

“Bluett RJ, Gamble George JC, Hermanson DJ, Hartley ND, Marnett LJ, Patel S. Central anandamide deficiency predictions stress induced anxiety: behavioral reverse through endocannabinoid augmentation. *Trans Psychology*. (2014) 4(7):e408. doi: 10.1038/tp.2014.53

Briânis RC, Andreotti JP, Moreira FA, Iglesias LP. Interplay between endocannabinoid and endovanilloid mechanisms in fear conditioning. *Acta Neuropsychiatr*. (2024) 36(5):255-264. doi: 10.1017/neu.2023.54

Burstein O, Shoshan N, Doron R, Akirav I. Cannabinoids prevent depressive-like symptoms and alterations in BDNF expression in a rat model of PTSD. *Prog Neuropsychopharmacol Biol Psychiatry*. (2018) 84(Pt A):129-139. doi: 10.1016/j.pnpbp.2018.01.026

Diniz CRAF, Biojone C, Joca SRL, Rantamäki T, Castrén E, Guimarães FS, et al. dual mechanism of TrkB activation by anandamide through CB1 and TRPV1 receptors. *peerj*. (2019) 7:e6493. doi: 10.7717/peerj.6493

Guimarães MEA, Derhon V, Signori LU, Seiffer BA, Wolf S, Schuch FB. Acute and chronic effects of physical exercise in inflammatory biomarkers in people with depression: A systematic review with meta-analysis. *J Psychiatr Res*. (2024) 179:26–32 doi: 10.1016/j.jpsychires.2024.08.025

Hill JD, Zuluaga Ramirez V, Gajgrate S, Winfield M, Persisky Y. activation of GPR55 increases neural stem cell promotion and promotions early adult hippocampal neurogenesis. *Br J pharmacol*. (2018) 175(16):3407-3421. doi: 10.1111/bph.14387

Hill MN, Hillard CJ, Bambico FR, Patel S, Gorzalka BB, Gobbi G. The therapeutic potential of the endocannabinoid system for the development of a novel class of antidepressants. *Trends Pharmacol Sci*. (2009) 30(9):484-493. doi: 10.1016/j.tips.2009.06.006

Iannotti FA, Vitale rm. the endocannabinoid system and ppars: focus on their signaling crosstalk, action and translational regulation. *cells*. (2021) 10(3):586. doi: 10.3390/cells10030586.

Kapadia R, Yi JH, vemuganti R. mechanisms of anti-inflammatory and neuroprotective actions of PPAR gamma agonists. *front biosci*. (2008) 13:1813-1826. doi: 10.2741/2802.

Ribeiro MA, Aguiar RP, Scarante FF, et al. Spontaneous Activity of CB2 Receptors Attenuates Stress-Induced Behavioral and Neuroplastic Deficits in Male Mice. *Front Pharmacol*. (2022) 12:805758. doi: 10.3389/fphar.2021.805758

Raichlen Da, Foster ad, German GL, Seillier a, Giuffrida A. wired to run: exercise induced endocannabinoid signaling in humans and cursorial matrices with implications for the ‘runner’s High’. *J exp biol*. (2012) 215(PT 8):1331-1336. doi: 10.1242/jeb.063677

Rodrigues RS, Moreira JB, Dias P, Sebastião AM, Xapelli S. The Effects of Exercise-Associated Factors on Hippocampal Progenitor Cell Dynamics Are Mediated by Cannabinoid Type 2 Receptors. *J Neurochem*. (2025) 169(5):e70091. doi:10.1111/jnc.70091

Rodrigues RS, Moreira JB, Mateus JM, et al. Cannabinoid type 2 receptor inhibition enhances the antidepressant and proneurogenic effects of physical exercise after chronic stress. *Transl Psychiatry*. (2024) 14(1):170. doi: 10.1038/s41398-024-02877-0

Sylantyev S, Jensen TP, Ross RA, Rusakov Da. Cannabinoid- and lysophosphatidylinositol sensitive receptor GPR55 boosts neurotransmitter release at central synapses. *proc Natl Acad SCI USA*. (2013) 110 (13): 5193-5198. doi:1 0.1073/pnas.1211204110”.

In the published article, “Rodrigues RS, Moreira JB, Mateus JM, et al. Cannabinoid type 2 receptor inhibition enhances the antidepressant and proneurogenic effects of physical exercise after chronic stress. Transl Psychiatry. 2024;14(1):170. Published 2024 Mar 30. doi:10.1038/s41398-024-02877-0”, a study on physical exercise after chronic stress, was erroneously cited where the sentence referred to a conventional SSRI antidepressant. A corrections has been made to **5 The potential of exercise and the ECS in the treatment of depression**, *5.4.2 Evidence contradictions and measurement limitations of ECS as an intermediary biomarker*, Paragraph 2:

“The complexity of ECS involvement is further exemplified by findings that the spontaneous activity of CB2 receptors is specifically required for the antidepressant-like and pro-neurogenic effects, but not the anxiolytic effects, of a conventional SSRI antidepressant in stressed mice (Ribeiro et al., 2022)”

The original version of this article has been updated.

